# Supplementary material for: Protocol for a multicentre, prospective observational cohort study in Japan: association among hospital-acquired disability, regular exercise and long-term care dependency in older patients after cardiac surgery
Source: BMJ Open. 2021 Dec 13;11(12):e054303. doi: 10.1136/bmjopen-2021-054303 (PMC8671971; doi:10.1136/bmjopen-2021-054303)
Supplement: Author's manuscript [file bmjopen-2021-054303.draft_revisions.pdf]

BMJ Open is committed to open peer review. As part of this commitment we make the peer review history of every article we publish publicly available.

When an article is published we post the peer reviewers' comments and the authors' responses online. We also post the versions of the paper that were used during peer review. These are the versions that the peer review comments apply to.

The versions of the paper that follow are the versions that were submitted during the peer review process. They are not the versions of record or the final published versions. They should not be cited or distributed as the published version of this manuscript.

BMJ Open is an open access journal and the full, final, typeset and author-corrected version of record of the manuscript is available on our site with no access controls, subscription charges or pay-per-view fees (http://bmjopen.bmj.com).

If you have any questions on BMJ Open's open peer review process please email info.bmjopen@bmj.com

BMJ Open

Protocol for a multicentre, prospective observational cohort study in Japan: association among hospital-acquired functional disability, regular exercise, and long-term care dependency in older patients after cardiac surgery

Journal:	*BMJ Open*
Manuscript ID	bmjopen-2021-054303
Article Type:	Protocol
Date Submitted by the Author:	09-Jun-2021
Complete List of Authors:	Saitoh, Masakazu; Juntendo University, Department of Physical Therapy, Faculty of Health Science<br>Takahashi, Tetsuya; Juntendo University, Department of Physical Therapy, Faculty of Health Science<br>Morisawa, Tomoyuki; Juntendo University, Department of Physical Therapy, Faculty of Health Science<br>Sakuyama, Akihiro; Juntendo University, Department of Physical Therapy, Faculty of Health Science<br>Watanabe, Hidetaka; Juntendo University Hospital, Department of Rehabilitation<br>Sakurada, Koji; The Cardiovascular Institute, Department of Rehabilitation<br>Hanafusa, Yusuke; Saitama Medical University International Medical Center, Department of Rehabilitation<br>Tahara, Masayuki; Higashitakarazuka Sato Hospital, Department of Physical therapy<br>Iwata, Kentaro; Kobe City Medical Center General Hospital, Department of Rehabilitation<br>Ochi, Yusuke; Fukuyama Cardiovascular Hospital, Department of Rehabilitation<br>Takamura, Go; Tsuchiya General Hospital, Department of Rehabilitation<br>Minei, Akira; Ryukyu University, Department of Rehabilitation
Keywords:	Breast surgery < SURGERY, REHABILITATION MEDICINE, GERIATRIC MEDICINE

SCHOLARONE™
Manuscripts

I, the Submitting Author has the right to grant and does grant on behalf of all authors of the Work (as defined in the below author licence), an exclusive licence and/or a non-exclusive licence for contributions from authors who are: i) UK Crown employees; ii) where BMJ has agreed a CC-BY licence shall apply, and/or iii) in accordance with the terms applicable for US Federal Government officers or employees acting as part of their official duties; on a worldwide, perpetual, irrevocable, royalty-free basis to BMJ Publishing Group Ltd ("BMJ") its licensees and where the relevant Journal is co-owned by BMJ to the co-owners of the Journal, to publish the Work in this journal and any other BMJ products and to exploit all rights, as set out in our licence.

The Submitting Author accepts and understands that any supply made under these terms is made by BMJ to the Submitting Author unless you are acting as an employee on behalf of your employer or a postgraduate student of an affiliated institution which is paying any applicable article publishing charge ("APC") for Open Access articles. Where the Submitting Author wishes to make the Work available on an Open Access basis (and intends to pay the relevant APC), the terms of reuse of such Open Access shall be governed by a Creative Commons licence – details of these licences and which Creative Commons licence will apply to this Work are set out in our licence referred to above.

Other than as permitted in any relevant BMJ Author's Self Archiving Policies, I confirm this Work has not been accepted for publication elsewhere, is not being considered for publication elsewhere and does not duplicate material already published. I confirm all authors consent to publication of this Work and authorise the granting of this licence.

For peer review only

Protocol for a multicentre, prospective observational cohort study in Japan: association among hospital-acquired functional disability, regular exercise, and long-term care dependency in older patients after cardiac surgery

Masakazu Saitoh[1], Tetsuya Takahashi[1], Tomoyuki Morisawa[1], Akihiro Sakuyama[1], Hidetaka Watanabe[2], Koji Sakurada[3], Yusuke Hanafusa[4], Masayuki Tahara[5], Kentaro Iwata[6], Yusuke Ochi[7], Go Takamura[8], Akira Minei[9]

1. Department of Physical Therapy, Faculty of Health Science, Juntendo University, Tokyo, Japan
2. Department of Rehabilitation, Juntendo University Hospital, Tokyo, Japan
3. Department of Rehabilitation, The Cardiovascular Institute, Tokyo, Japan
4. Rehabilitation Center, Saitama Medical University International Medical Center, Hidaka, Saitama, Japan
5. Department of Physical therapy, Higashitakarazuka Sato Hospital, Takarazuka, Hyogo, Japan
6. Department of Rehabilitation, Kobe City Medical Center General Hospital, Kobe, Hyogo, Japan
7. Department of Rehabilitation, Fukuyama Cardiovascular Hospital, Fukuyama, Hiroshima, Japan
8. Department of Rehabilitation, Tsuchiya General Hospital, Hiroshima, Hiroshima, Japan
9. Department of Rehabilitation, Ryukyu University, Nishihara-cho, Okinawa, Japan

Word count: 2607 words

Keywords: protocol, hospital-acquired functional disability, long-term care dependency

Corresponding author
Tetsuya Takahashi, RPT, PhD
Department of Physical Therapy,
Faculty of Health Science, Juntendo University
e-mail: te-takahashi@juntendo.ac.jp

For peer review only

ABSTRACT

Introduction

Prevention of functional decline and long-term care dependency after cardiac surgery is important; however, our current understanding of postoperative functional trajectory and effects of postoperative regular exercise on long-term functional decline and long-term care dependency is limited. Therefore, we will conduct a multicentre, prospective cohort study to 1) examine the effect of hospital-acquired disability (HAD) on long-term functional decline and long-term care dependency and 2) investigate the favourable effect of postoperative regular exercise on long-term functional decline and long-term care dependency in older patients after cardiac surgery.

Methods and analysis

We designed a prospective, multicentre cohort study to enrol older patients aged ≥65 years undergoing elective coronary artery bypass graft or valve surgery. We will conduct medical record reviews to collect data on patient demographics, comorbidities, operative details, progression of in-hospital postoperative cardiac rehabilitation, and functional trajectory from a few days before cardiac surgery to the day before hospital discharge. They will be followed up for 2 years to obtain information on their health status including functional status, regular exercise, and clinical events by mail. Primary endpoints of this study are long-term functional decline and long-term care dependency after cardiac surgery. Secondary endpoints are readmission due to cardiac events or all-cause mortality. We will evaluate whether a certain HAD pattern predicts and whether regular exercise prevents post-discharge functional decline and long-term care dependency.

Ethics and dissemination

The study protocol was approved by the ethics committee of the Department of Physical Therapy, Faculty of Health Science, Juntendo University, and of each collaborating hospital. We obtained written informed consent from all study participants after the description of the study procedures. The informed consent form states that all clinical information is confidential and will not be shared and that data are stored in a secure web-based database (REDCap). Patient recruitment began in April 2021, and the 2-year follow-up is expected to be completed in 2024. Publication of the study results is anticipated in 2025.

Strength and limitations of this study

For peer review only

- A prospective, multicentre cohort study will be conducted to investigate the impact of hospital acquired disability (HAD) on functional decline and long-term care dependency.
- The study will identify risk factors of HAD in older patients undergoing elective cardiac surgery.
- The study involves a detailed prospective follow-up after discharge to ascertain functional status, regular exercise, and rehospitalisation due to cardiac events and all-cause mortality.
- An important potential limitation is selection bias introduced by the inclusion of individuals who meet the eligibility criteria and are theoretically capable of functional assessment or answering the questionnaire on functional status and regular exercise, as well as those with severe cognitive impairment. This may lead to the increased likelihood of other risk factors of functional decline and long-term care dependency and clinical events.

For peer review only

INTRODUCTION

Japan is now categorised as a super-aged society, with a population of individuals aged ≥65 years being 35.89 million (28.4%),[1] which will reach 30% in 2025 and 40% in 2060. [2] This has led to an increase in the number of older patients undergoing cardiac surgery, despite the higher risk of postoperative complications and short-term mortality in older patients.[3] In the consideration of cardiac surgery for older patients, functional decline and the need for long-term care have received increasing attention as essential outcomes in recent years.

Hospital-acquired disability (HAD) or functional decline, which refers to either a new or worsening disability or functional decline during hospitalisation not present before hospitalisation, develops in 25–50% of hospitalised elderly patients. [4,5] In-hospital complications or comorbidities could lead to a longer hospitalisation stay after cardiac surgery. [6-8] Although the impact of HAD on long-term functional decline and long-term care dependency may be significant, it has not been investigated in detail yet.

Most studies have not prospectively measured functional trajectory from baseline to hospital discharge and long-term follow-up. [9,10] In addition, the need for long-term care, which is defined as a condition wherein older patients need care to perform activities of daily living (ADLs), increases with age and disease. The number of individuals who need long-term care in Japan has increased, leading to increasing social security expense for long-term care. [11] Thus, the Japanese government launched a policy with the aim to extend healthy life expectancy without the need for long-term care. In Japan, long-term care is required mainly for patients with dementia (18.7%), cerebrovascular disease (15.1%), age-related frailty (13.8%), falls and fractures (12.5%), joint disease (10.2%), and cardiac disease (4.7).(1) However, the trajectory for long-term care requirement after cardiac surgery remains unclear.

Recent meta-analyses showed that exercise-based cardiac rehabilitation (CR) improves exercise capacity in patients after cardiac surgery. [12,13] However, CR implementation rate for patients with acute myocardial infarction and heart failure is very low (21% and 7%, respectively). [14,15] In particular, there are few reports on CR implementation rate in older patients with frailty or functional disability after cardiac surgery. [16] However, several previous studies have demonstrated that CR or geriatric rehabilitation improves functional status and quality of life in older patients with frailty or functional decline. [17,18] We hypothesised that functional trajectory after cardiac surgery and regular exercise after hospital discharge significantly impact

For peer review only

long-term functional decline and long-term care dependency as well as morbidity and mortality; however, this hypothesis has not been tested yet.

In this context, we developed a multicentre prospective cohort study for elderly patients undergoing cardiac surgery. The primary objective of the present study is to prospectively investigate the association of functional trajectory after cardiac surgery and post-discharge regular exercise with clinical outcomes. The primary outcome of the present study are long-term functional decline and long-term care dependency. Secondary outcomes are hospitalisation, and all-cause mortality.

METHODS AND ANALYSIS

Study design

The study is a multicentre, prospective cohort study in Japan enrolling older patients awaiting elective cardiac surgery at hospital admission; these patients will be followed up for 2 years after discharge. The overall study design is outlined in Figure 1.

Participant eligibility criteria

Potential participants are recruited from collaborating hospitals. To be eligible for study participation, patients must be aged ≥65 years and scheduled to undergo elective cardiac surgery. Exclusion criteria are presented in Figure 2. All patients provided informed consent at the time of recruitment.

Collaborating hospitals

All collaborating hospitals provided a standardised in-hospital CR program according to the Guidelines of Japanese Association of Cardiac Rehabilitation. [19] Functional assessments are performed by a trained physiotherapist according to the functional assessment manual.

Measurements

The collaborating hospitals are encouraged to register the patients as consecutively as possible. Once informed consent is obtained, patients are assigned a study ID. For each patient, demographic data, aetiology of cardiac surgery, comorbidities, laboratory data, echocardiographic findings, medications, and progression of postoperative in-hospital CR are collected. A mail survey on clinical outcomes and questionnaire on frailty, functional status, and regular exercise (home-based non-supervised exercise, centre-based geriatric rehabilitation, centre-based CR) will be conducted at 6, 12, and 24 months after hospital discharge.

For peer review only

Activity of daily living

The baseline Barthel index (BI) was evaluated on the basis of interviews with patients or family members, depending on the patient's cognitive function, approximately 1 month before hospital admission, as in a previous study. [20] BI was evaluated by a trained physiotherapist at each hospital. BI is a simple 10-item instrument that measures functional independence in ADLs. The total score ranges from 0 to 100 points, with higher scores indicating greater independence in performing basic ADLs. HAD was defined as a decrease by at least 5 points on BI the day before discharge to home, nursing care facility, or other hospitals compared with preadmission BI. Sub-analyses were performed using no HAD, mild HAD as 5-point BI score decrease, and severe HAD as ≥10-point BI score decrease. [20]

Frailty assessment

The Kihon Checklist (KCL), a self-reported comprehensive health checklist, is a screening tool used to identify community-dwelling older adults who are vulnerable to frailty and at greater risk of becoming dependent. [21] The KCL consists of 25 items (yes/no) divided into seven categories: physical strength, nutrition, eating, socialisation, memory, mood, and lifestyle; each item was scored from 0 to 1 point, and the sum of all indices can be 0–25; a total score of ≥8 points is classified as indicating frailty. In addition, seven specific domains of frailty—instrumental ADL, physical, nutrition, oral, socialisation, memory, and mood domains—were scored.

The Japanese version of the Cardiovascular Health Study (J-CHS) criteria was constructed by modifying and simplifying the original CHS criteria to be suited to Japanese older adults. [22] Moreover, J-CHS can predict a new occurrence of long-term care need and loss of independence. It consists of 5 items: 1) Shrinking "Have you unintentionally lost 2 kg or more in the past 6 months?" 2) Low activity a) "Do you engage in moderate levels of physical exercise or sports aimed at health?" b) Do you engage in low levels of physical exercise aimed at health?" 3) Exhaustion "In the past 2 weeks, have you felt tired without a reason?" 4) Weakness "defined as grip strength <28 kg in men and 18 kg in women," 5) Slowness "defined as gait speed of <1.0 m/s." Each item was scored as 0 or 1 point, and all points were added to calculate total score. A total score of ≥3 points was considered as indicating frailty.

Short Physical Performance Battery

The short physical performance battery (SPPB) is a highly standardised geriatric physical functioning test that consists of tests for balance, gait, strength, and endurance. [23] The standing balance test includes side-by-side, semi-tandem, and tandem standing, and patients are timed until they lose balance or 10 s elapses. The gait test assesses the time taken to walk 4 m and is performed at patient's usual pace. The 5-time chair-standing test, a pre-test, is also performed, wherein patients are asked to fold their arms across their chest and stand up from the chair. If the pre-test is successful, patients are asked to rise from the chair 5 times consecutively as quickly as possible. Each of the three subtests of SPPB is scored from 0 to 4 and summed for an SPPB score of 0–12 points, with higher scores indicating better physical function.

Hand grip strength

Isometric hand grip strength is measured using the Jamar hand dynamometer, which measures strength in kilograms with a precision of 0.1 kg. Patients are instructed to sit on a straight-backed chair with feet placed flat on the floor, shoulder adducted and neutrally rotated, elbow flexed at 90°, and forearm and wrist in neutral position, as recommended by Roberts et al. Grip strength is measured twice per hand in each of the two handle positions. [24] Patients are instructed to apply maximum power for 3 s and work at maximum effort in every trial.

In-hospital postoperative cardiac rehabilitation

The acute phase of the CR program following cardiovascular surgery has been described elsewhere[8] and is based on the Japanese Circulation Society guidelines for rehabilitation of patients with cardiovascular disease. [19] We, therefore, define failure to achieve early ambulation as ≥3 postoperative days taken to initiate walking exercise according to the guidelines. Moreover, the proportion of patients who can complete a 100-m corridor walk without assistance at a comfortable pace within 3 days of hospital admission is investigated; early ambulation is an important indicator of mobility or physical activity recovery during hospitalisation. [25]

Outcome measures

The primary endpoints of this study are functional status trajectory and long-term care dependency. Functional status is measured using the KCL total score and specific 7-domain score. Secondary endpoints are readmissions because of cardiac events and all-cause mortality.

For peer review only

Sample size calculation

Sample size is calculated by performing multivariate analysis, which examines the relationship among functional trajectory, regular exercise, physical activity, and clinical outcomes. Eight independent variables, including functional trajectory, regular exercise, and known prognostic factors of patients undergoing cardiac surgery, are selected for multivariate analysis. Assuming 10 outcomes per independent variable, 80 outcomes need to be observed. Based on our preliminary data, 8% incidence of a cardiac event in 2 years was estimated. With a 10% missing follow-up data, the necessary sample size was calculated to be 1100 patients. Because we also plan to perform a cross-sectional analysis using baseline data, a preliminary sample size calculation was done. Although the association of functional trajectory after cardiac surgery with risk factors related clinical outcomes including morbidity, mortality, functional decline, and long-term care. We estimated that HAD incidence in the group with delayed in-hospital CR was 30%, compared with 20% in the group with normal progression of the guideline-based CR. A sample size of 1092 patients was estimated to be necessary based on the statistical power of 80% at a significance level of 5%. Thus, the estimated required number of patients in the cross-sectional analysis is sufficient for the prospective analysis. Therefore, we set the overall sample size as 1100 in the present study.

Statistical analysis

Statistical analysis will be performed to identify risk factors of functional trajectory after cardiac surgery. The resulting dataset is a complex time-series data, with each patient having 3 data points (preoperative, POD 7, and hospital discharge) for each physical performance measures. The trajectories can be labelled according to the observed clinical phenotype as below: complete recovery, partial recovery, and ADL dependence in each patient with and without physical frailty. This data-driven classification allows the identification of predictors in patients belonging to a certain class of functional trajectory path. To identify predictors of functional trajectories, multinominal logistic regression models will be applied using preoperative demographics, comorbidities, peri- and postoperative variables, and baseline functional status or frailty. Multivariate Cox regression analysis will be used to separately model the risk of (1) 2-year functional decline, (2) 2-year long-term care dependency level, (3) 2-year all-cause mortality, and (4) 2-year cardiovascular event.

Data statement section

All patient data are stored in a secure web-based database (REDCap) with limited access and ID code to which data are directly transferred. Patients are assigned unique identification numbers, which are the only identifiers exported from REDcap during data analysis (Figure 3). The principal investigator has access to all data. Data can be accessed on request to the corresponding author after reports related to the primary outcomes are published.

Patient confidentiality

The present study was organised to according to the Guidelines for Epidemiological Research proposed by the Japanese Ministry of Health, Labour and Welfare. The study protocol was approved by the ethics committee of the Department of Physical Therapy, Juntendo University (approval no. 20-030). Ethical approval was also obtained from each collaborating hospital. Each patient provided written informed consent to be registered in this study.

Patient and public involvement

Patients and/or the public were not involved in the design, or conduct, or reporting, or dissemination plans of this research.

Strength and limitations

A primary outcome of older patients after cardiac surgery that receives special focus is morbidity or mortality; however, more recently, healthy life expectancy and quality-adjusted life years are also becoming important targets of treatment. Functional status or long-term care are, therefore, recognised as essential outcomes for promoting informed surgical decision for patients with cardiovascular diseases, their family, and the surgeon. Despite extensive investigations into morbidity or mortality, very few studies have specifically investigated the risk of functional decline and long-term care dependency following cardiovascular surgery. To our knowledge, the present study is the first large-scale, prospective, multicentre cohort study to examine the association of functional trajectory during hospitalisation and post-discharge regular exercise with functional decline and long-term care dependency. This study may suggest novel strategies to prevent functional decline and long-term care dependency in older patients after cardiac surgery.

The prognostic impact of HAD has recently been suggested in community-dwelling older adults or older patients with heart failure. However, the effect of HAD on long-term functional decline and long-term care dependency remains unknown.

For peer review only

Moreover, another purpose of this cohort study is to examine the effects of regular exercise including CR on functional decline and long-term care dependency. In particular, exercise-based CR has favourable effects on functional status in older patients with poor functional status. Our large cohort study allows us to analyse the effects of outpatient CR on functional trajectory in patients with HAD.

In addition, physical as well as psychological and social aspects are important to maintain healthy life expectancy or quality-adjusted life years in older patients. Cognitive decline or decline in the social aspect may aid in the assessment of functional decline and long-term care dependency. Based on these findings, we assessed physical status, cognitive status, and social status from KCL subcomponent scores.

ETHICS AND DISSEMINATION

The study protocol was approved by the ethics committee of the Department of Physical Therapy, Faculty of Health Science, Juntendo University, and of each collaborating hospital. We obtained written informed consent from all study participants after the description of the study procedures. The informed consent form states that all clinical information is confidential and will not be shared and that data are stored in a secure web-based database (REDCap). Patient recruitment began in April 2021, and the 2-year follow-up is expected to be completed in 2024. The study results will be published in a peer-reviewed journal, in addition to being presented at national and international conferences. We anticipate these results to be published in 2024, after completion of the 2-year follow-up of all recruited patients. Our findings will help inform future recommendations of postoperative care planning for older patients after cardiac surgery.

This study has an important potential limitation is selection bias introduced by the inclusion of individuals who meet the eligibility criteria and are theoretically capable of functional assessment or answering the questionnaire on functional status and regular exercise, as well as those with severe cognitive impairment. This may lead to the increased likelihood of other risk factors of functional decline and long-term care dependency and clinical events.

CONCLUSION

The results from this cohort study will help patients and health providers in better stratification of patients at risk of long-term functional decline and long-term care

For peer review only

dependency after cardiac surgery and improve postoperative care planning for older patients undergoing cardiac surgery.

Authors' contributions

MS, TM, and TT conceived the study and obtained grant funding. MS, TM, and TT drafted the protocol. AM, AS, GT, HW, KI, KS, MT, YH, and YO reviewed and revised the protocol.

Funding

This work is supported by the MEXT KAKENHI Grant Number JP 20H04055.

Competing interests

None declared.

References

1. The Cabinet Office, government of Japan. Annual Report on the Ageing Society: 2018.Available: https://www8.cao.go.jp/kourei/whitepaper/index-w.html [Accessed at 15 May 2021].

2. World Population Prospects: The 2019 revision: United Nations, 2019. Available: https://population.un.org/wpp/ [Accessed at 15 May 2021].

3. Porizka M, Kunstyr J, Vanek T, et al. Postoperative outcome of high-risk octogenarians undergoing cardiac surgery: A multicenter observational retrospective study. *Ann Thorac Cardiovasc Surg* 2017;23:188–95.

4. Satake S, Senda K, Hong YJ, et al. Validity of the Kihon Checklist for assessing frailty status. *Geriatr Gerontol Int* 2016;16:709–15.

5. Covinsky KE, Pierluissi E, Johnston CB. Hospitalization-associated disability: "She was probably able to ambulate, but I'm not sure". *JAMA* 2011;306:1782–93.

6. LaPar DJ, Speir AM, Crosby IK, et al. Postoperative atrial fibrillation significantly increases mortality, hospital readmission, and hospital costs. *Ann Thorac Surg* 2014;98:527–33.

7. Hollenbeak CS, Murphy DM, Koenig S, et al. The clinical and economic impact of deep chest surgical site infections following coronary artery bypass graft surgery. *Chest* 2000;118:397–402.

For peer review only

8. Saitoh M, Takahashi T, Sakurada K, et al. Factors determining achievement of early postoperative cardiac rehabilitation goal in patients with or without preoperative kidney dysfunction undergoing isolated cardiac surgery. *J Cardiol* 2013;61:299–303.

9. Nakano M, Nomura Y, Suffredini G, et al. Functional outcomes of frail patients after cardiac surgery: An observational study. *Anesth Analg* 2020;130:1534–44.

10. Gaulton TG, Neuman MD. Association between obesity, age, and functional decline in survivors of cardiac surgery. *J Am Geriatr Soc* 2018;66:127–32.

11. Tsutsui T. The current state and future development of the long-term care insurance system in Japan. *Journal of the National Institute of Public Health* 2010;59:372–9.

12. Abraham LN, Sibilitz KL, Berg SK, et al. Exercise-based cardiac rehabilitation for adults after heart valve surgery. *Cochrane Database Syst Rev* 2021;5:Cd010876.

13. Doyle MP, Indraratna P, Tardo DT, et al. Safety and efficacy of aerobic exercise commenced early after cardiac surgery: A systematic review and meta-analysis. *Eur J Prev Cardiol* 2019;26:36–45.

14. Arakawa T, Kumasaka L, Nakanishi M, et al. Regional clinical alliance path and cardiac rehabilitation after hospital discharge for acute myocardial infarction patients in Japan- A nationwide survey. *Circ J* 2016;80:1750–5.

15. Kamiya K, Yamamoto T, Tsuchihashi-Makaya M, et al. Nationwide survey of multidisciplinary care and cardiac rehabilitation for patients with heart failure in Japan- An analysis of the AMED-CHF study. *Circ J* 2019;83:1546–52.

16. Tamulevičiūtė-Prascienė E, Beigienė A, Thompson MJ, Balnė K, Kubilius R, Bjarnason-Wehrens B. The impact of additional resistance and balance training in exercise-based cardiac rehabilitation in older patients after valve surgery or intervention: randomized control trial. *BMC Geriatr* 2021;21:23.

17. Zanettini R, Gatto G, Mori I, et al. Cardiac rehabilitation and mid-term follow-up after transcatheter aortic valve implantation. *J Geriatr Cardiol* 2014;11:279–85.

18. Kitzman DW, Whellan DJ, Duncan P, et al. Physical rehabilitation for older patients hospitalized for heart failure. *N Engl J Med* 2021. doi: 10.1056/NEJMoa2026141.

19. JCS/JACR 2021 Guidelines on rehabilitation in patients with cardiovascular disease. Available: https://www.j-circ.or.jp/cms/wp-content/uploads/2021/03/JCS2021_Makita.pdf ［Accessed at 15 May 2021］.

20. Saitoh M, Takahashi Y, Okamura D, et al. Prognostic impact of hospital-acquired disability in elderly patients with heart failure. *ESC Heart Fail* 2021;8:1767–74.

For peer review only

21. Satake S, Shimokata H, Senda K, et al. Validity of total Kihon checklist score for predicting the incidence of 3-year dependency and mortality in a community-dwelling older population. *J Am Med Dir Assoc* 2017;18:552.e1–.e6.
22. Satake S, Shimada H, Yamada M, et al. Prevalence of frailty among community-dwellers and outpatients in Japan as defined by the Japanese version of the Cardiovascular Health Study criteria. *Geriatr Gerontol Int* 2017;17:2629–34.
23. Guralnik JM, Simonsick EM, Ferrucci L, et al. A short physical performance battery assessing lower extremity function: association with self-reported disability and prediction of mortality and nursing home admission. *J Gerontol* 1994;49:M85–94.
24. Roberts HC, Denison HJ, Martin HJ, et al. A review of the measurement of grip strength in clinical and epidemiological studies: towards a standardised approach. *Age Ageing* 2011;40:423–9.
25. Saioth M, Kamisaka K, Kato M, et al. Effect of obesity and underweight status on the hospital-acquired functional decline in patients with cardiovascular surgery: an analysis of data from a prospective observational multicenter cohort study. *Gen Thorac Cardiovasc Surg* 2020;68:914–21.

For peer review only

Figure legends

Figure 1: Experimental protocol

Figure 2: Overall design of the study and main objectives

Figure 3: Data registration system and database security

For peer review only

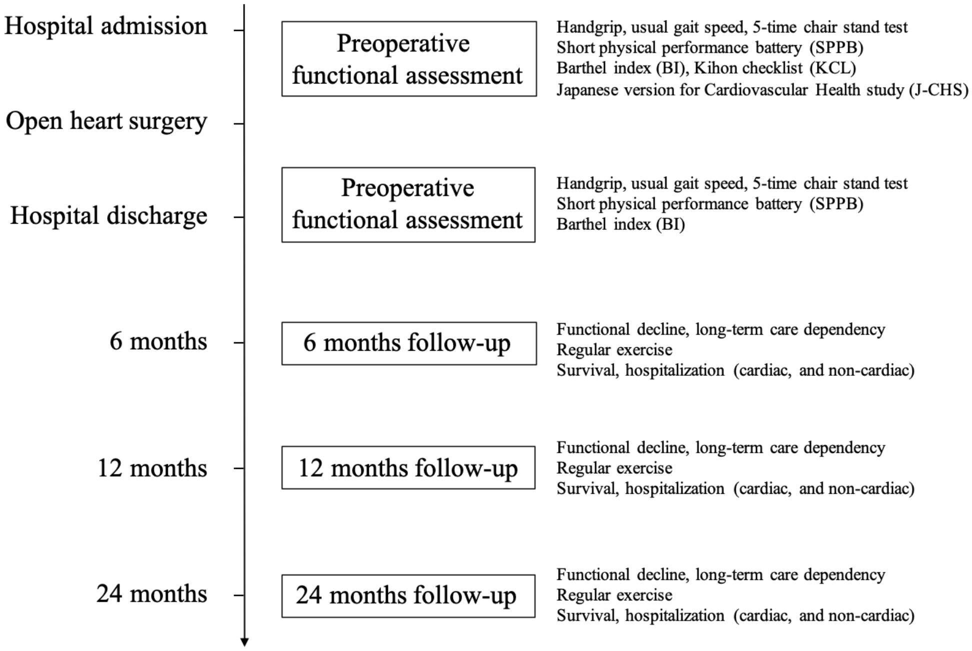

Figure 1

482x317mm (72 x 72 DPI)

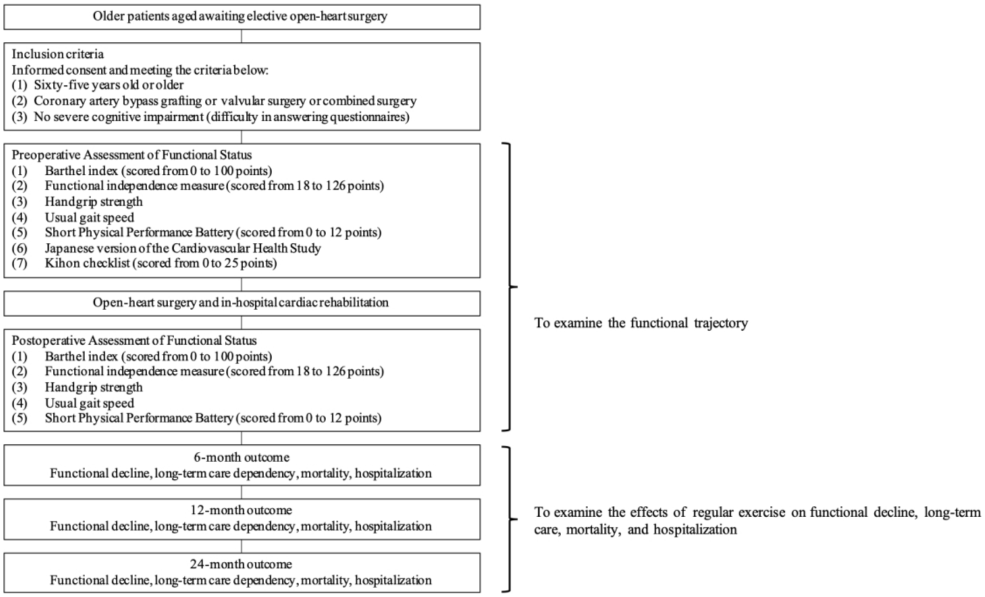

Figure 2

573x340mm (72 x 72 DPI)

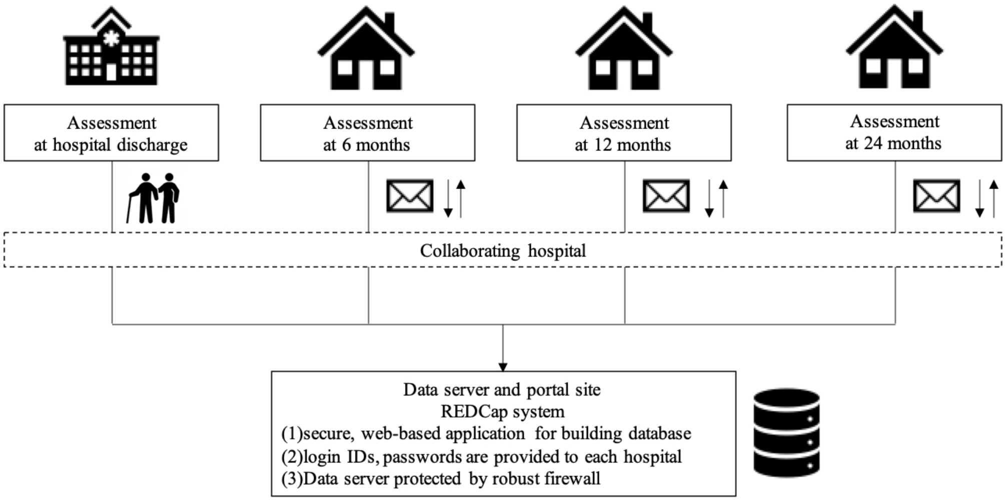

Figure 3

451x230mm (72 x 72 DPI)

STANDARD PROTOCOL ITEMS: RECOMMENDATIONS FOR INTERVENTIONAL TRIALS

SPIRIT 2013 Checklist: Recommended items to address in a clinical trial protocol and related documents*

Section/item	Item No	Description
**Administrative information**		
Title	1	Descriptive title identifying the study design, population, interventions, and, if applicable, trial acronym
Trial registration	2a	Trial identifier and registry name. If not yet registered, name of intended registry
	2b	All items from the World Health Organization Trial Registration Data Set
Protocol version	3	Date and version identifier
Funding	4	Sources and types of financial, material, and other support
Roles and responsibilities	5a	Names, affiliations, and roles of protocol contributors
	5b	Name and contact information for the trial sponsor
	5c	Role of study sponsor and funders, if any, in study design; collection, management, analysis, and interpretation of data; writing of the report; and the decision to submit the report for publication, including whether they will have ultimate authority over any of these activities
	5d	Composition, roles, and responsibilities of the coordinating centre, steering committee, endpoint adjudication committee, data management team, and other individuals or groups overseeing the trial, if applicable (see Item 21a for data monitoring committee)
**Introduction**		
Background and rationale	6a	Description of research question and justification for undertaking the trial, including summary of relevant studies (published and unpublished) examining benefits and harms for each intervention
	6b	Explanation for choice of comparators
Objectives	7	Specific objectives or hypotheses
Trial design	8	Description of trial design including type of trial (eg, parallel group, crossover, factorial, single group), allocation ratio, and framework (eg, superiority, equivalence, noninferiority, exploratory)

For peer review only

Methods: Participants, interventions, and outcomes

Study setting	9	Description of study settings (eg, community clinic, academic hospital) and list of countries where data will be collected. Reference to where list of study sites can be obtained
Eligibility criteria	10	Inclusion and exclusion criteria for participants. If applicable, eligibility criteria for study centres and individuals who will perform the interventions (eg, surgeons, psychotherapists)
Interventions	11a	Interventions for each group with sufficient detail to allow replication, including how and when they will be administered
	11b	Criteria for discontinuing or modifying allocated interventions for a given trial participant (eg, drug dose change in response to harms, participant request, or improving/worsening disease)
	11c	Strategies to improve adherence to intervention protocols, and any procedures for monitoring adherence (eg, drug tablet return, laboratory tests)
	11d	Relevant concomitant care and interventions that are permitted or prohibited during the trial
Outcomes	12	Primary, secondary, and other outcomes, including the specific measurement variable (eg, systolic blood pressure), analysis metric (eg, change from baseline, final value, time to event), method of aggregation (eg, median, proportion), and time point for each outcome. Explanation of the clinical relevance of chosen efficacy and harm outcomes is strongly recommended
Participant timeline	13	Time schedule of enrolment, interventions (including any run-ins and washouts), assessments, and visits for participants. A schematic diagram is highly recommended (see Figure)
Sample size	14	Estimated number of participants needed to achieve study objectives and how it was determined, including clinical and statistical assumptions supporting any sample size calculations
Recruitment	15	Strategies for achieving adequate participant enrolment to reach target sample size

Methods: Assignment of interventions (for controlled trials)

Allocation:

Sequence generation	16a	Method of generating the allocation sequence (eg, computer-generated random numbers), and list of any factors for stratification. To reduce predictability of a random sequence, details of any planned restriction (eg, blocking) should be provided in a separate document that is unavailable to those who enrol participants or assign interventions

For peer review only - http://bmjopen.bmj.com/site/about/guidelines.xhtml

Allocation concealment mechanism	16b	Mechanism of implementing the allocation sequence (eg, central telephone; sequentially numbered, opaque, sealed envelopes), describing any steps to conceal the sequence until interventions are assigned
Implementation	16c	Who will generate the allocation sequence, who will enrol participants, and who will assign participants to interventions
Blinding (masking)	17a	Who will be blinded after assignment to interventions (eg, trial participants, care providers, outcome assessors, data analysts), and how
	17b	If blinded, circumstances under which unblinding is permissible, and procedure for revealing a participant's allocated intervention during the trial
**Methods: Data collection, management, and analysis**		
Data collection methods	18a	Plans for assessment and collection of outcome, baseline, and other trial data, including any related processes to promote data quality (eg, duplicate measurements, training of assessors) and a description of study instruments (eg, questionnaires, laboratory tests) along with their reliability and validity, if known. Reference to where data collection forms can be found, if not in the protocol
	18b	Plans to promote participant retention and complete follow-up, including list of any outcome data to be collected for participants who discontinue or deviate from intervention protocols
Data management	19	Plans for data entry, coding, security, and storage, including any related processes to promote data quality (eg, double data entry; range checks for data values). Reference to where details of data management procedures can be found, if not in the protocol
Statistical methods	20a	Statistical methods for analysing primary and secondary outcomes. Reference to where other details of the statistical analysis plan can be found, if not in the protocol
	20b	Methods for any additional analyses (eg, subgroup and adjusted analyses)
	20c	Definition of analysis population relating to protocol non-adherence (eg, as randomised analysis), and any statistical methods to handle missing data (eg, multiple imputation)
**Methods: Monitoring**		
Data monitoring	21a	Composition of data monitoring committee (DMC); summary of its role and reporting structure; statement of whether it is independent from the sponsor and competing interests; and reference to where further details about its charter can be found, if not in the protocol. Alternatively, an explanation of why a DMC is not needed

For peer review only - http://bmjopen.bmj.com/site/about/guidelines.xhtml

	21b	Description of any interim analyses and stopping guidelines, including who will have access to these interim results and make the final decision to terminate the trial
Harms	22	Plans for collecting, assessing, reporting, and managing solicited and spontaneously reported adverse events and other unintended effects of trial interventions or trial conduct
Auditing	23	Frequency and procedures for auditing trial conduct, if any, and whether the process will be independent from investigators and the sponsor
**Ethics and dissemination**		
Research ethics approval	24	Plans for seeking research ethics committee/institutional review board (REC/IRB) approval
Protocol amendments	25	Plans for communicating important protocol modifications (eg, changes to eligibility criteria, outcomes, analyses) to relevant parties (eg, investigators, REC/IRBs, trial participants, trial registries, journals, regulators)
Consent or assent	26a	Who will obtain informed consent or assent from potential trial participants or authorised surrogates, and how (see Item 32)
	26b	Additional consent provisions for collection and use of participant data and biological specimens in ancillary studies, if applicable
Confidentiality	27	How personal information about potential and enrolled participants will be collected, shared, and maintained in order to protect confidentiality before, during, and after the trial
Declaration of interests	28	Financial and other competing interests for principal investigators for the overall trial and each study site
Access to data	29	Statement of who will have access to the final trial dataset, and disclosure of contractual agreements that limit such access for investigators
Ancillary and post-trial care	30	Provisions, if any, for ancillary and post-trial care, and for compensation to those who suffer harm from trial participation
Dissemination policy	31a	Plans for investigators and sponsor to communicate trial results to participants, healthcare professionals, the public, and other relevant groups (eg, via publication, reporting in results databases, or other data sharing arrangements), including any publication restrictions
	31b	Authorship eligibility guidelines and any intended use of professional writers
	31c	Plans, if any, for granting public access to the full protocol, participant-level dataset, and statistical code

For peer review only - http://bmjopen.bmj.com/site/about/guidelines.xhtml

Appendices

Informed consent materials	32	Model consent form and other related documentation given to participants and authorised surrogates
Biological specimens	33	Plans for collection, laboratory evaluation, and storage of biological specimens for genetic or molecular analysis in the current trial and for future use in ancillary studies, if applicable

*It is strongly recommended that this checklist be read in conjunction with the SPIRIT 2013 Explanation & Elaboration for important clarification on the items. Amendments to the protocol should be tracked and dated. The SPIRIT checklist is copyrighted by the SPIRIT Group under the Creative Commons "Attribution-NonCommercial-NoDerivs 3.0 Unported" license.

For peer review only

BMJ Open

Protocol for a multicentre, prospective observational cohort study in Japan: association among hospital-acquired disability, regular exercise, and long-term care dependency in older patients after cardiac surgery

Journal:	*BMJ Open*
Manuscript ID	bmjopen-2021-054303.R1
Article Type:	Protocol
Date Submitted by the Author:	11-Nov-2021
Complete List of Authors:	Saitoh, Masakazu; Juntendo University, Department of Physical Therapy, Faculty of Health Science<br>Takahashi, Tetsuya; Juntendo University, Department of Physical Therapy, Faculty of Health Science<br>Morisawa, Tomoyuki; Juntendo University, Department of Physical Therapy, Faculty of Health Science<br>Sakuyama, Akihiro; Juntendo University, Department of Physical Therapy, Faculty of Health Science<br>Watanabe, Hidetaka; Juntendo University Hospital, Department of Rehabilitation<br>Sakurada, Koji; The Cardiovascular Institute, Department of Rehabilitation<br>Hanafusa, Yusuke; Saitama Medical University International Medical Center, Department of Rehabilitation<br>Tahara, Masayuki; Higashitakarazuka Sato Hospital, Department of Physical therapy<br>Iwata, Kentaro; Kobe City Medical Center General Hospital, Department of Rehabilitation<br>Ochi, Yusuke; Fukuyama Cardiovascular Hospital, Department of Rehabilitation<br>Takamura, Go; Tsuchiya General Hospital, Department of Rehabilitation<br>Minei, Akira; Ryukyu University, Department of Rehabilitation
**Primary Subject Heading**:	Rehabilitation medicine
Secondary Subject Heading:	Cardiovascular medicine
Keywords:	CARDIOLOGY, GERIATRIC MEDICINE, REHABILITATION MEDICINE

SCHOLARONE™
Manuscripts

I, the Submitting Author has the right to grant and does grant on behalf of all authors of the Work (as defined in the below author licence), an exclusive licence and/or a non-exclusive licence for contributions from authors who are: i) UK Crown employees; ii) where BMJ has agreed a CC-BY licence shall apply, and/or iii) in accordance with the terms applicable for US Federal Government officers or employees acting as part of their official duties; on a worldwide, perpetual, irrevocable, royalty-free basis to BMJ Publishing Group Ltd ("BMJ") its licensees and where the relevant Journal is co-owned by BMJ to the co-owners of the Journal, to publish the Work in this journal and any other BMJ products and to exploit all rights, as set out in our licence.

The Submitting Author accepts and understands that any supply made under these terms is made by BMJ to the Submitting Author unless you are acting as an employee on behalf of your employer or a postgraduate student of an affiliated institution which is paying any applicable article publishing charge ("APC") for Open Access articles. Where the Submitting Author wishes to make the Work available on an Open Access basis (and intends to pay the relevant APC), the terms of reuse of such Open Access shall be governed by a Creative Commons licence – details of these licences and which Creative Commons licence will apply to this Work are set out in our licence referred to above.

Other than as permitted in any relevant BMJ Author's Self Archiving Policies, I confirm this Work has not been accepted for publication elsewhere, is not being considered for publication elsewhere and does not duplicate material already published. I confirm all authors consent to publication of this Work and authorise the granting of this licence.

For peer review only

Protocol for a multicentre, prospective observational cohort study in Japan: association among hospital-acquired disability, regular exercise, and long-term care dependency in older patients after cardiac surgery

Masakazu Saitoh[1], Tetsuya Takahashi[1], Tomoyuki Morisawa[1], Akihiro Sakuyama[1], Hidetaka Watanabe[2], Koji Sakurada[3], Yusuke Hanafusa[4], Masayuki Tahara[5], Kentaro Iwata[6], Yusuke Ochi[7], Go Takamura[8], Akira Minei[9]

1. Department of Physical Therapy, Faculty of Health Science, Juntendo University, Tokyo, Japan
2. Department of Rehabilitation, Juntendo University Hospital, Tokyo, Japan
3. Department of Rehabilitation, The Cardiovascular Institute, Tokyo, Japan
4. Rehabilitation Center, Saitama Medical University International Medical Center, Hidaka, Saitama, Japan
5. Department of Physical therapy, Higashitakarazuka Sato Hospital, Takarazuka, Hyogo, Japan
6. Department of Rehabilitation, Kobe City Medical Center General Hospital, Kobe, Hyogo, Japan
7. Department of Rehabilitation, Fukuyama Cardiovascular Hospital, Fukuyama, Hiroshima, Japan
8. Department of Rehabilitation, Tsuchiya General Hospital, Hiroshima, Hiroshima, Japan
9. Department of Rehabilitation, Ryukyu University, Nishihara-cho, Okinawa, Japan

Word count: 2607 words

Keywords: protocol, hospital-acquired disability, long-term care dependency

Corresponding author
Tetsuya Takahashi, RPT, PhD
Department of Physical Therapy,
Faculty of Health Science, Juntendo University
e-mail: te-takahashi@juntendo.ac.jp

For peer review only - http://bmjopen.bmj.com/site/about/guidelines.xhtml

ABSTRACT

Introduction

Cardiac surgery for older patients, postoperative functional decline and the need for long-term care have received increasing attention as essential outcomes in recent years. It is therefore prevention of functional decline and long-term care dependency after cardiac surgery is important; however, our current understanding of postoperative functional trajectory and effects of postoperative regular exercise on long-term functional decline and long-term care dependency is limited. Therefore, we will conduct a multicentre, prospective cohort study to 1) examine the effect of hospital-acquired disability (HAD) on long-term functional decline and long-term care dependency and 2) investigate the favourable effect of postoperative regular exercise on long-term functional decline and long-term care dependency in older patients after cardiac surgery.

Methods and analysis

We designed a prospective, multicentre cohort study to enrol older patients aged ≥65 years undergoing elective coronary artery bypass graft or valve surgery. We will conduct medical record reviews to collect data on patient demographics, comorbidities, operative details, progression of in-hospital postoperative cardiac rehabilitation, and functional trajectory from a few days before cardiac surgery to the day before hospital discharge. They will be followed up for 2 years to obtain information on their health status including functional status, regular exercise, and clinical events by mail. Primary endpoints of this study are long-term functional decline and long-term care dependency after cardiac surgery. Secondary endpoints are readmission due to cardiac events or all-cause mortality.

Ethics and dissemination

The study protocol was approved by the ethics committee of the Department of Physical Therapy, Faculty of Health Science, Juntendo University, and of each collaborating hospital. We obtained written informed consent from all study participants after the description of the study procedures. Publication of the study results is anticipated in 2025.

For peer review only - http://bmjopen.bmj.com/site/about/guidelines.xhtml

Strength and limitations of this study

- As the first prospective, multicentre cohort study will provide important evidence on the trajectory of the functional status, long-term care dependency in older patients undergoing elective cardiac surgery.
- Risk factors for Hospital acquired disability in older patients undergoing elective cardiac surgery will be investigated.
- Prospective follow-up will help to identify new strategy associated with trajectory of the long-term care dependency and long-term health status.
- This study is not population based due to the recruitment scheme in collaborative hospital that focuses on research and education in the field of cardiac surgery rehabilitation, which limits its generalisability and underestimate the prevalence of functional decline.
- An important potential limitation is selection bias introduced by the inclusion of individuals who meet the eligibility criteria and are capable of functional assessments or answering the questionnaires on functional status.

For peer review only

For peer review only - http://bmjopen.bmj.com/site/about/guidelines.xhtml

INTRODUCTION

Japan is now categorised as a super-aged society, with a population of individuals aged ≥65 years being 35.89 million (28.4%),[1] which will reach 30% in 2025 and 40% in 2060. [2] The Japanese Association for Thoracic Surgery has conducted annual surveys of thoracic surgery throughout Japan. Cardiac surgery is the most common of thoracic surgery, more than 70,000 patients in Japan undergo cardiac surgery annually [3], in particularly the absolute number of older patients undergoing cardiac surgery is increasing.[4] In the consideration of cardiac surgery for older patients, functional decline and the need for long-term care have received increasing attention as essential outcomes in recent years.

Hospital-acquired disability (HAD) or functional decline, which refers to either a new or worsening disability or functional decline during hospitalisation not present before hospitalisation, develops in 25–50% of hospitalised elderly patients. [5,6] In-hospital complications or comorbidities could lead to a longer hospitalisation stay after cardiac surgery. [7-9] Although the impact of HAD on long-term functional decline and long-term care dependency may be significant, it has not been investigated in detail yet.

Most studies have not prospectively measured functional trajectory from baseline to hospital discharge and long-term follow-up. [10,11] In addition, the need for long-term care, which is defined as a condition wherein older patients need care to perform activities of daily living (ADLs), increases with age and disease. The number of individuals who need long-term care in Japan has increased, leading to increasing social security expense for long-term care. [12] Thus, the Japanese government launched a policy with the aim to extend healthy life expectancy without the need for long-term care. In Japan, long-term care is required mainly for patients with dementia (18.7%), cerebrovascular disease (15.1%), age-related frailty (13.8%), falls and fractures (12.5%), joint disease (10.2%), and cardiac disease (4.7).(1) However, the trajectory for long-term care requirement after cardiac surgery remains unclear.

Recent meta-analyses showed that exercise-based cardiac rehabilitation (CR) improves exercise capacity in patients after cardiac surgery. [13,14] However, CR implementation rate for patients with acute myocardial infarction and heart failure is very low (21% and 7%, respectively). [15,16] In particular, there are few reports on CR implementation rate in older patients with frailty or functional disability after cardiac surgery. [17] However, several previous studies have demonstrated that CR or geriatric rehabilitation improves functional status and quality of life in older patients with frailty or functional decline. [18,19] We hypothesised that functional trajectory

For peer review only - http://bmjopen.bmj.com/site/about/guidelines.xhtml

after cardiac surgery and regular exercise after hospital discharge significantly impact long-term functional decline and long-term care dependency as well as morbidity and mortality; however, this hypothesis has not been tested yet.

In this context, we developed a multicentre prospective cohort study for elderly patients undergoing cardiac surgery. The primary objective of the present study is to prospectively investigate the association of functional trajectory after cardiac surgery and post-discharge regular exercise with clinical outcomes. The primary outcome of the present study are long-term functional decline and long-term care dependency. Secondary outcomes are hospitalisation, and all-cause mortality.

METHODS AND ANALYSIS

Study design and setting

The study is a multicentre, prospective cohort study that will be carried out between August 2021 and December 2024 in Japan enrolling older patients awaiting elective cardiac surgery at hospital admission; these patients will be followed up for 2 years after discharge. The overall study design is outlined in Figure 1.

Participant eligibility criteria

Potential participants are recruited from collaborating hospitals. To be eligible for study participation, patients must be aged ≥65 years and scheduled to undergo elective cardiac surgery. Exclusion criteria are presented in Figure 2. All patients provided informed consent at the time of recruitment.

Collaborating hospitals

All collaborating hospitals provided a standardised in-hospital CR program according to the Guidelines of Japanese Association of Cardiac Rehabilitation. [20] Functional assessments are performed by a trained physiotherapist according to the functional assessment manual.

Measurements

The collaborating hospitals are encouraged to register the patients as consecutively as possible. Once informed consent is obtained, patients are assigned a study ID. For each patient, demographic data, aetiology of cardiac surgery, comorbidities, laboratory data, echocardiographic findings, medications, and progression of postoperative in-hospital CR are collected. A mail survey on clinical outcomes and questionnaire on frailty, functional status, and regular exercise (home-based non-supervised exercise,

For peer review only - http://bmjopen.bmj.com/site/about/guidelines.xhtml

centre-based geriatric rehabilitation, centre-based CR) will be conducted at 6, 12, and 24 months after hospital discharge.

Activity of daily living

The baseline Barthel index (BI) was evaluated on the basis of interviews with patients or family members, depending on the patient's cognitive function, approximately 1 month before hospital admission, as in a previous study. [21] BI was evaluated by a trained physiotherapist at each hospital. BI is a simple 10-item instrument that measures functional independence in ADLs. The total score ranges from 0 to 100 points, with higher scores indicating greater independence in performing basic ADLs. HAD was defined as a decrease by at least 5 points on BI the day before discharge to home, nursing care facility, or other hospitals compared with preadmission BI. Sub-analyses were performed using no HAD, mild HAD as 5-point BI score decrease, and severe HAD as ≥10-point BI score decrease. [21]

Frailty assessment

The Kihon Checklist (KCL), a self-reported comprehensive health checklist, is a screening tool used to identify community-dwelling older adults who are vulnerable to frailty and at greater risk of becoming dependent. [22] The KCL consists of 25 items (yes/no) divided into seven categories: physical strength, nutrition, eating, socialisation, memory, mood, and lifestyle; each item was scored from 0 to 1 point, and the sum of all indices can be 0–25; a total score of ≥8 points is classified as indicating frailty. In addition, seven specific domains of frailty—instrumental ADL, physical, nutrition, oral, socialisation, memory, and mood domains—were scored.

The Japanese version of the Cardiovascular Health Study (J-CHS) criteria was constructed by modifying and simplifying the original CHS criteria to be suited to Japanese older adults. [23] Moreover, J-CHS can predict a new occurrence of long-term care need and loss of independence. It consists of 5 items: 1) Shrinking "Have you unintentionally lost 2 kg or more in the past 6 months?" 2) Low activity a) "Do you engage in moderate levels of physical exercise or sports aimed at health?" b) Do you engage in low levels of physical exercise aimed at health?" 3) Exhaustion "In the past 2 weeks, have you felt tired without a reason?" 4) Weakness "defined as grip strength <28 kg in men and 18 kg in women," 5) Slowness "defined as gait speed of <1.0 m/s." Each item was scored as 0 or 1 point, and all points were added to calculate total score. A total score of ≥3 points was considered as indicating frailty.

For peer review only - http://bmjopen.bmj.com/site/about/guidelines.xhtml

Short Physical Performance Battery

The short physical performance battery (SPPB) is a highly standardised geriatric physical functioning test that consists of tests for balance, gait, strength, and endurance. [24] The standing balance test includes side-by-side, semi-tandem, and tandem standing, and patients are timed until they lose balance or 10 s elapses. The gait test assesses the time taken to walk 4 m and is performed at patient's usual pace. The 5-time chair-standing test, a pre-test, is also performed, wherein patients are asked to fold their arms across their chest and stand up from the chair. If the pre-test is successful, patients are asked to rise from the chair 5 times consecutively as quickly as possible. Each of the three subtests of SPPB is scored from 0 to 4 and summed for an SPPB score of 0–12 points, with higher scores indicating better physical function.

Hand grip strength

Isometric hand grip strength is measured using the Jamar hand dynamometer, which measures strength in kilograms with a precision of 0.1 kg. Patients are instructed to sit on a straight-backed chair with feet placed flat on the floor, shoulder adducted and neutrally rotated, elbow flexed at 90°, and forearm and wrist in neutral position, as recommended by Roberts et al. Grip strength is measured twice per hand in each of the two handle positions. [25] Patients are instructed to apply maximum power for 3 s and work at maximum effort in every trial.

In-hospital postoperative cardiac rehabilitation

The acute phase of the CR program following cardiovascular surgery has been described elsewhere[9] and is based on the Japanese Circulation Society guidelines for rehabilitation of patients with cardiovascular disease. [20] We, therefore, define failure to achieve early ambulation as ≥3 postoperative days taken to initiate walking exercise according to the guidelines. Moreover, the proportion of patients who can complete a 100-m corridor walk without assistance at a comfortable pace within 3 days of hospital admission is investigated; early ambulation is an important indicator of mobility or physical activity recovery during hospitalisation. [26]

Outcome measures

The primary endpoints of this study are functional status trajectory and long-term care dependency. Functional status is measured using the KCL total score and specific 7-domain score. Secondary endpoints are readmissions because of cardiac events and all-cause mortality.

For peer review only - http://bmjopen.bmj.com/site/about/guidelines.xhtml

Sample size calculation

Sample size is calculated by performing multivariate analysis, which examines the relationship among functional trajectory, regular exercise, physical activity, and clinical outcomes. Eight independent variables, including functional trajectory, regular exercise, and known prognostic factors of patients undergoing cardiac surgery, are selected for multivariate analysis. Assuming 10 outcomes per independent variable, 80 outcomes need to be observed. Based on our preliminary data, 8% incidence of a cardiac event in 2 years was estimated. With a 10% missing follow-up data, the necessary sample size was calculated to be 1100 patients. Because we also plan to perform a cross-sectional analysis using baseline data, a preliminary sample size calculation was done. Although the association of functional trajectory after cardiac surgery with risk factors related clinical outcomes including morbidity, mortality, functional decline, and long-term care. We estimated that HAD incidence in the group with delayed in-hospital CR was 30%, compared with 20% in the group with normal progression of the guideline-based CR. A sample size of 1092 patients was estimated to be necessary based on the statistical power of 80% at a significance level of 5%. Thus, the estimated required number of patients in the cross-sectional analysis is sufficient for the prospective analysis. Therefore, we set the overall sample size as 1100 in the present study.

Statistical analysis

Continuous variables will be presented through centrality measures (mean or median), and dispersion (Standard deviation or interquartile range), and categorical variables through frequencies and percentages. Statistical analysis will be performed to identify risk factors of functional trajectory after cardiac surgery. The resulting dataset is a complex time-series data, with each patient having 3 data points (preoperative, POD 7, and hospital discharge) for each physical performance measures. The trajectories can be labelled according to the observed clinical phenotype as below: complete recovery, partial recovery, and ADL dependence in each patient with and without physical frailty. This data-driven classification allows the identification of predictors in patients belonging to a certain class of functional trajectory path. To identify predictors of functional trajectories, multinominal logistic regression models will be applied using preoperative demographics, comorbidities, peri- and postoperative variables, and baseline functional status or frailty. Multivariate Cox regression analysis will be used to separately model the risk of (1) 2-year functional decline, (2) 2-year long-term care dependency level, (3) 2-year all-cause mortality, and (4) 2-year cardiovascular event.

For peer review only - http://bmjopen.bmj.com/site/about/guidelines.xhtml

Missing data on endpoints will not be replaced. A replacement method, multiple imputations through chained equations will be envisaged for other variables and other variables. A *P*-value <0.05 will be considered statistically significant.

Data statement section

All patient data are stored in a secure web-based database (REDCap) with limited access and ID code to which data are directly transferred. Patients are assigned unique identification numbers, which are the only identifiers exported from REDcap during data analysis (Figure 3). The principal investigator has access to all data. Data can be accessed on request to the corresponding author after reports related to the primary outcomes are published.

Patient confidentiality

The present study was organised to according to the Guidelines for Epidemiological Research proposed by the Japanese Ministry of Health, Labour and Welfare. The study protocol was approved by the ethics committee of the Department of Physical Therapy, Juntendo University (approval no. 20-030). Ethical approval was also obtained from each collaborating hospital. Each patient provided written informed consent to be registered in this study.

Patient and public involvement

Patients and/or the public were not involved in the design, or conduct, or reporting, or dissemination plans of this research.

Strength and limitations

A primary outcome of older patients after cardiac surgery that receives special focus is morbidity or mortality; however, more recently, healthy life expectancy and quality-adjusted life years are also becoming important targets of treatment. Functional status or long-term care are, therefore, recognised as essential outcomes for promoting informed surgical decision for patients with cardiovascular diseases, their family, and the surgeon. Despite extensive investigations into morbidity or mortality, very few studies have specifically investigated the risk of functional decline and long-term care dependency following cardiovascular surgery. To our knowledge, the present study is the first large-scale, prospective, multicentre cohort study to examine the association of functional trajectory during hospitalisation and post-discharge regular exercise with

For peer review only - http://bmjopen.bmj.com/site/about/guidelines.xhtml

functional decline and long-term care dependency. This study may suggest novel strategies to prevent functional decline and long-term care dependency in older patients after cardiac surgery.

The prognostic impact of HAD has recently been suggested in community-dwelling older adults or older patients with heart failure. However, the effect of HAD on long-term functional decline and long-term care dependency remains unknown. Moreover, another purpose of this cohort study is to examine the effects of regular exercise including CR on functional decline and long-term care dependency. In particular, exercise-based CR has favourable effects on functional status in older patients with poor functional status. Our large cohort study allows us to analyse the effects of outpatient CR on functional trajectory in patients with HAD.

In addition, physical as well as psychological and social aspects are important to maintain healthy life expectancy or quality-adjusted life years in older patients. Cognitive decline or decline in the social aspect may aid in the assessment of functional decline and long-term care dependency. Based on these findings, we assessed physical status, cognitive status, and social status from KCL subcomponent scores.

This study is not population based due to the recruitment scheme in the collaborative hospitals that focuses on research and education in the field of cardiac surgery rehabilitation. Thus, this study is limited in its generalisability and underestimates the prevalence of functional decline. However, all facilities performed a large number of cardiac surgeries on older patients, normative older populations are also included in the study. Another limitation is selection bias introduced by the inclusion of individuals who meet the eligibility criteria and are capable of functional assessments or answering the questionnaires on functional status.

ETHICS AND DISSEMINATION

The study protocol was approved by the ethics committee of the Department of Physical Therapy, Faculty of Health Science, Juntendo University, and of each collaborating hospital. We obtained written informed consent from all study participants after the description of the study procedures. The informed consent form states that all clinical information is confidential and will not be shared and that data are stored in a secure web-based database (REDCap). Patient recruitment began in April 2021, and the 2-year follow-up is expected to be completed in 2024. The study results will be published in a peer-reviewed journal, in addition to being presented at national and international conferences. We anticipate these results to be published in 2024, after completion of the 2-year follow-up of all recruited patients. Our findings will help

For peer review only - http://bmjopen.bmj.com/site/about/guidelines.xhtml

inform future recommendations of postoperative care planning for older patients after cardiac surgery.

This study has an important potential limitation is selection bias introduced by the inclusion of individuals who meet the eligibility criteria and are theoretically capable of functional assessment or answering the questionnaire on functional status and regular exercise, as well as those with severe cognitive impairment. This may lead to the increased likelihood of other risk factors of functional decline and long-term care dependency and clinical events.

Authors' contributions

MS, TM, and TT conceived the study and obtained grant funding. MS, TM, and TT drafted the protocol. AM, AS, GT, HW, KI, KS, MT, YH, and YO reviewed and revised the protocol.

Funding

This work is supported by the MEXT KAKENHI Grant Number JP 20H04055.

Competing interests

None declared.

References

1. The Cabinet Office, government of Japan. Annual Report on the Ageing Society: 2018.Available: https://www8.cao.go.jp/kourei/whitepaper/index-w.html [Accessed at 15 May 2021].
2. World Population Prospects: The 2019 revision: United Nations, 2019. Available: https://population.un.org/wpp/ [Accessed at 15 May 2021].
3. Shimizu H, Okada M, Tangoku A, et al. Thoracic and cardiovascular surgeries in Japan during 2017 : Annual report by the Japanese Association for Thoracic Surgery. *Gen Thorac Cardiovasc Surg.* 2020;68(4):414-449.
4. Porizka M, Kunstyr J, Vanek T, et al. Postoperative outcome of high-risk octogenarians undergoing cardiac surgery: A multicenter observational retrospective study. *Ann Thorac Cardiovasc Surg* 2017;23:188–95.
5. Satake S, Senda K, Hong YJ, et al. Validity of the Kihon Checklist for assessing frailty status. *Geriatr Gerontol Int* 2016;16:709–15.

For peer review only - http://bmjopen.bmj.com/site/about/guidelines.xhtml

6. Covinsky KE, Pierluissi E, Johnston CB. Hospitalization-associated disability: "She was probably able to ambulate, but I'm not sure". *JAMA* 2011;306:1782–93.

7. LaPar DJ, Speir AM, Crosby IK, et al. Postoperative atrial fibrillation significantly increases mortality, hospital readmission, and hospital costs. *Ann Thorac Surg* 2014;98:527–33.

8. Hollenbeak CS, Murphy DM, Koenig S, et al. The clinical and economic impact of deep chest surgical site infections following coronary artery bypass graft surgery. *Chest* 2000;118:397–402.

9. Saitoh M, Takahashi T, Sakurada K, et al. Factors determining achievement of early postoperative cardiac rehabilitation goal in patients with or without preoperative kidney dysfunction undergoing isolated cardiac surgery. *J Cardiol* 2013;61:299–303.

10. Nakano M, Nomura Y, Suffredini G, et al. Functional outcomes of frail patients after cardiac surgery: An observational study. *Anesth Analg* 2020;130:1534–44.

11. Gaulton TG, Neuman MD. Association between obesity, age, and functional decline in survivors of cardiac surgery. *J Am Geriatr Soc* 2018;66:127–32.

12. Tsutsui T. The current state and future development of the long-term care insurance system in Japan. *Journal of the National Institute of Public Health* 2010;59:372–9.

13. Abraham LN, Sibilitz KL, Berg SK, et al. Exercise-based cardiac rehabilitation for adults after heart valve surgery. *Cochrane Database Syst Rev* 2021;5:Cd010876.

14. Doyle MP, Indraratna P, Tardo DT, et al. Safety and efficacy of aerobic exercise commenced early after cardiac surgery: A systematic review and meta-analysis. *Eur J Prev Cardiol* 2019;26:36–45.

15. Arakawa T, Kumasaka L, Nakanishi M, et al. Regional clinical alliance path and cardiac rehabilitation after hospital discharge for acute myocardial infarction patients in Japan- A nationwide survey. *Circ J* 2016;80:1750–5.

16. Kamiya K, Yamamoto T, Tsuchihashi-Makaya M, et al. Nationwide survey of multidisciplinary care and cardiac rehabilitation for patients with heart failure in Japan- An analysis of the AMED-CHF study. *Circ J* 2019;83:1546–52.

17. Tamulevičiūtė-Prascienė E, Beigienė A, Thompson MJ, Balnė K, Kubilius R, Bjarnason-Wehrens B. The impact of additional resistance and balance training in exercise-based cardiac rehabilitation in older patients after valve surgery or intervention: randomized control trial. *BMC Geriatr* 2021;21:23.

18. Zanettini R, Gatto G, Mori I, et al. Cardiac rehabilitation and mid-term follow-up after transcatheter aortic valve implantation. *J Geriatr Cardiol* 2014;11:279–85.

For peer review only - http://bmjopen.bmj.com/site/about/guidelines.xhtml

19. Kitzman DW, Whellan DJ, Duncan P, et al. Physical rehabilitation for older patients hospitalized for heart failure. *N Engl J Med* 2021. doi: 10.1056/NEJMoa2026141.

20. JCS/JACR 2021 Guidelines on rehabilitation in patients with cardiovascular disease. Available: https://www.j-circ.or.jp/cms/wp-content/uploads/2021/03/JCS2021_Makita.pdf ［Accessed at 15 May 2021］.

21. Saitoh M, Takahashi Y, Okamura D, et al. Prognostic impact of hospital-acquired disability in elderly patients with heart failure. *ESC Heart Fail* 2021;8:1767–74.

22. Satake S, Shimokata H, Senda K, et al. Validity of total Kihon checklist score for predicting the incidence of 3-year dependency and mortality in a community-dwelling older population. *J Am Med Dir Assoc* 2017;18:552.e1–.e6.

23. Satake S, Shimada H, Yamada M, et al. Prevalence of frailty among community-dwellers and outpatients in Japan as defined by the Japanese version of the Cardiovascular Health Study criteria. *Geriatr Gerontol Int* 2017;17:2629–34.

24. Guralnik JM, Simonsick EM, Ferrucci L, et al. A short physical performance battery assessing lower extremity function: association with self-reported disability and prediction of mortality and nursing home admission. *J Gerontol* 1994;49:M85–94.

25. Roberts HC, Denison HJ, Martin HJ, et al. A review of the measurement of grip strength in clinical and epidemiological studies: towards a standardised approach. *Age Ageing* 2011;40:423–9.

26. Saioth M, Kamisaka K, Kato M, et al. Effect of obesity and underweight status on the hospital-acquired functional decline in patients with cardiovascular surgery: an analysis of data from a prospective observational multicenter cohort study. *Gen Thorac Cardiovasc Surg* 2020;68:914–21.

For peer review only

Figure legends

Figure 1: Experimental protocol

Figure 2: Overall design of the study and main objectives

Figure 3: Data registration system and database security

For peer review only

For peer review only - http://bmjopen.bmj.com/site/about/guidelines.xhtml

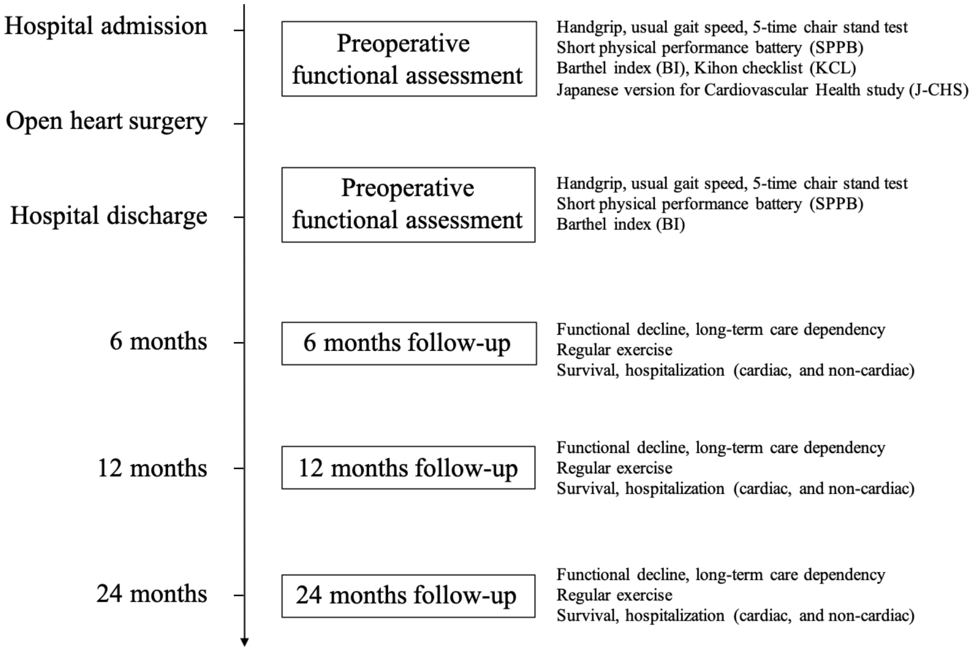

Figure 1

482x317mm (72 x 72 DPI)

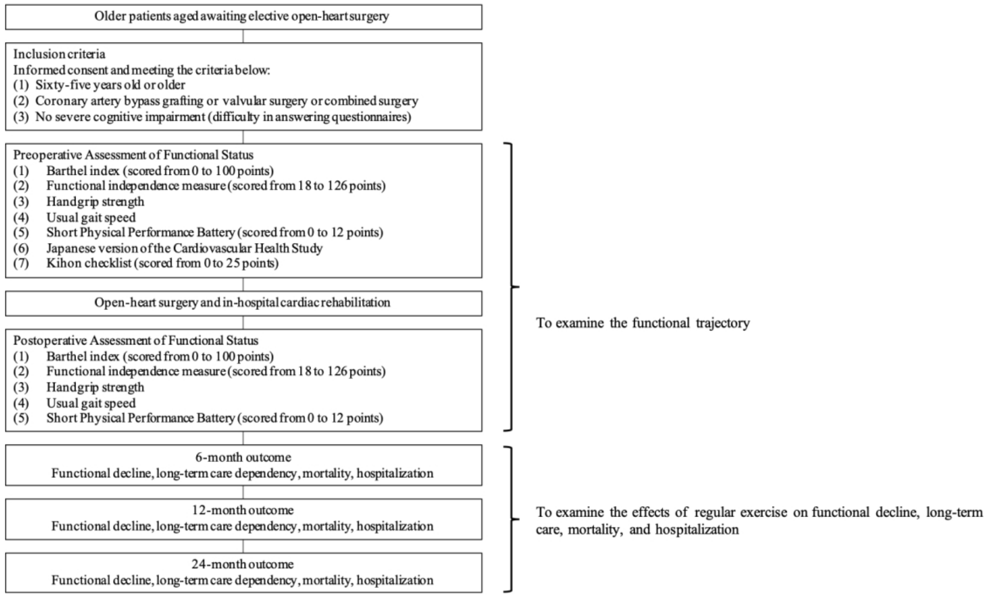

Figure 2

573x340mm (72 x 72 DPI)

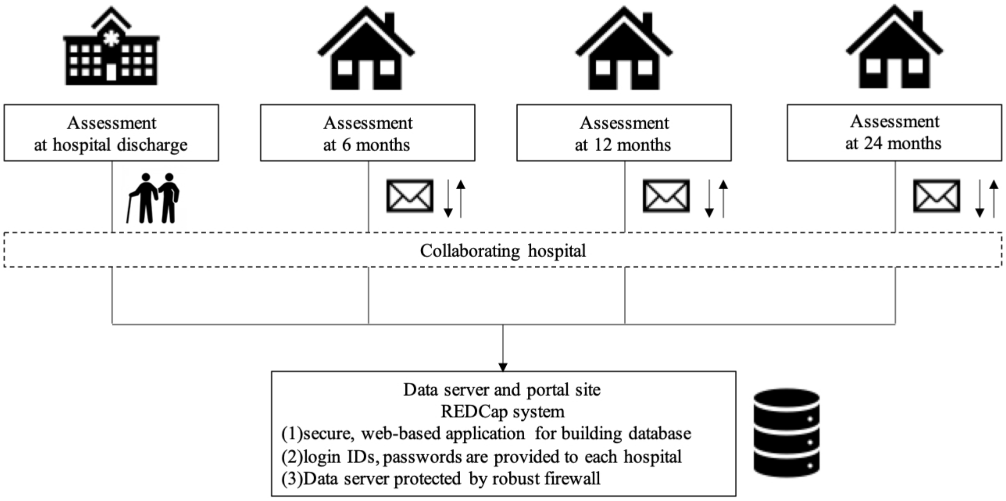

Figure 3

451x230mm (72 x 72 DPI)

STROBE Statement—Checklist of items that should be included in reports of ***cohort studies***

	Item No	Recommendation	Page, Line
**Title and abstract**	1	(*a*) Indicate the study's design with a commonly used term in the title or the abstract	Page 2, Line14-25
		(*b*) Provide in the abstract an informative and balanced summary of what was done and what was found	Page2, Line8-12
**Introduction**			
Background/rationale	2	Explain the scientific background and rationale for the investigation being reported	Page4, Line2-36
Objectives	3	State specific objectives, including any prespecified hypotheses	Page4, Line36 to Page5, Line3
**Methods**			
Study design	4	Present key elements of study design early in the paper	Page5, Line13-16
Setting	5	Describe the setting, locations, and relevant dates, including periods of recruitment, exposure, follow-up, and data collection	Page5, Line13-16
Participants	6	(*a*) Give the eligibility criteria, and the sources and methods of selection of participants. Describe methods of follow-up	Page5, Line19-22
		(*b*) For matched studies, give matching criteria and number of exposed and unexposed	n/a
Variables	7	Clearly define all outcomes, exposures, predictors, potential confounders, and effect modifiers. Give diagnostic criteria, if applicable	Page5, Line31 to Page, Line2
Data sources/ measurement	8*	For each variable of interest, give sources of data and details of methods of assessment (measurement). Describe comparability of assessment methods if there is more than one group	Page6, Line16 to Page7, Line20
Bias	9	Describe any efforts to address potential sources of bias	n/a
Study size	10	Explain how the study size was arrived at	Page8, Line3-19
Quantitative variables	11	Explain how quantitative variables were handled in the analyses. If applicable, describe which groupings were chosen and why	Page8, Line22-24
Statistical methods	12	(*a*) Describe all statistical methods, including those used to control for confounding	Page8, Line24 to Page, Line2
		(*b*) Describe any methods used to examine subgroups and interactions	Page8, Line31-34
		(*c*) Explain how missing data were addressed	Page9, Line1
		(*d*) If applicable, explain how loss to follow-up was addressed	Page9, Line1-3
		(*e*) Describe any sensitivity analyses	n/a

Results

For peer review only - http://bmjopen.bmj.com/site/about/guidelines.xhtml

For peer review only

Participants	13*	(a) Report numbers of individuals at each stage of study—eg numbers potentially eligible, examined for eligibility, confirmed eligible, included in the study, completing follow-up, and analysed	n/a
		(b) Give reasons for non-participation at each stage	n/a
		(c) Consider use of a flow diagram	n/a
Descriptive data	14*	(a) Give characteristics of study participants (eg demographic, clinical, social) and information on exposures and potential confounders	n/a
		(b) Indicate number of participants with missing data for each variable of interest	n/a
		(c) Summarise follow-up time (eg, average and total amount)	n/a
Outcome data	15*	Report numbers of outcome events or summary measures over time	n/a
Main results	16	(*a*) Give unadjusted estimates and, if applicable, confounder-adjusted estimates and their precision (eg, 95% confidence interval). Make clear which confounders were adjusted for and why they were included	n/a
		(*b*) Report category boundaries when continuous variables were categorized	n/a
		(*c*) If relevant, consider translating estimates of relative risk into absolute risk for a meaningful time period	n/a
Other analyses	17	Report other analyses done—eg analyses of subgroups and interactions, and sensitivity analyses	n/a
**Discussion**			
Key results	18	Summarise key results with reference to study objectives	n/a
Limitations	19	Discuss limitations of the study, taking into account sources of potential bias or imprecision. Discuss both direction and magnitude of any potential bias	Page10, Line16-23
Interpretation	20	Give a cautious overall interpretation of results considering objectives, limitations, multiplicity of analyses, results from similar studies, and other relevant evidence	n/a
Generalisability	21	Discuss the generalisability (external validity) of the study results	Page10, Line17-24
**Other information**			
Funding	22	Give the source of funding and the role of the funders for the present study and, if applicable, for the original study on which the present article is based	Page11, Line17

*Give information separately for exposed and unexposed groups.

Note: An Explanation and Elaboration article discusses each checklist item and gives methodological background and published examples of transparent reporting. The STROBE checklist is best used in conjunction with this article (freely available on the Web sites of PLoS Medicine at http://www.plosmedicine.org/, Annals of Internal Medicine at http://www.annals.org/, and Epidemiology at http://www.epidem.com/). Information on the STROBE Initiative is available at http://www.strobe-statement.org.

For peer review only - http://bmjopen.bmj.com/site/about/guidelines.xhtml
